# In memoriam: Professor Dan J. Stein (1962-2025) — a global pioneer with an African heart

**DOI:** 10.1093/ijnp/pyaf079

**Published:** 2025-12-18

**Authors:** Gregers Wegener, Bernard Lerer, Alan Frazer, Anthony Grace, Kazutaka Ikeda, Gabriella Gobbi, Allan Young, Joseph Zohar, Pierre Blier, Siegfried Kasper, John Krystal, Shigeto Yamawaki, Anthony Philips, Hans-Jürgen Möller, Robert H Belmaker, Lukoye Atwoli, David Nutt, Brian H Harvey, Soraya Seedat, Michael Berk, Robin Emsley

**Affiliations:** Department of Clinical Medicine, Aarhus University, Aarhus, Denmark; Department of Psychiatry, Hebrew University Hadassah Medical Center, Jerusalem, Israel; Department of Pharmacology, UT Health San Antonio, San Antonio, TX, United States; Departments of Neuroscience, Psychiatry & Psychology, University of Pittsburgh, Pittsburgh, PA, United States; Department of Psychiatry and Behavioral Sciences, Tokyo Metropolitan Institute of Medical Science, Tokyo, Japan; Department of Psychiatry, McGill University, Montreal, QC, Canada; Department of Brain Sciences, Imperial College London, London, United Kingdom; Department of Psychiatry, Tel Aviv University, Tel Aviv, Israel; Department of Psychiatry, University of Ottawa, Ottawa, ON, Canada; Department of Psychiatry and Psychotherapy, Medical University of Vienna, Vienna, Austria; Department of Psychiatry, Yale University, New Haven, CT, United States; Department of Psychiatry and Neurosciences, Hiroshima University, Hiroshima, Japan; Department of Psychiatry, University of British Columbia, Vancouver, BC, Canada; Department of Psychiatry, Ludwig-Maximilians-University, Munich, Germany; Department of Psychiatry, Ben-Gurion University of the Negev, Beersheva, Israel; Medical College, Aga Khan University, Nairobi, Kenya; Centre for Neuropsychopharmacology, Imperial College London, London, United Kingdom; Center of Excellence for Pharmaceutical Sciences, North-West University, Potchefstroom, South Africa; Department of Psychiatry, Stellenbosch University, Stellenbosch, South Africa; IMPACT Institute, Deakin University, Geelong/Melbourne, VIC, Australia; Department of Psychiatry, Stellenbosch University, Stellenbosch, South Africa


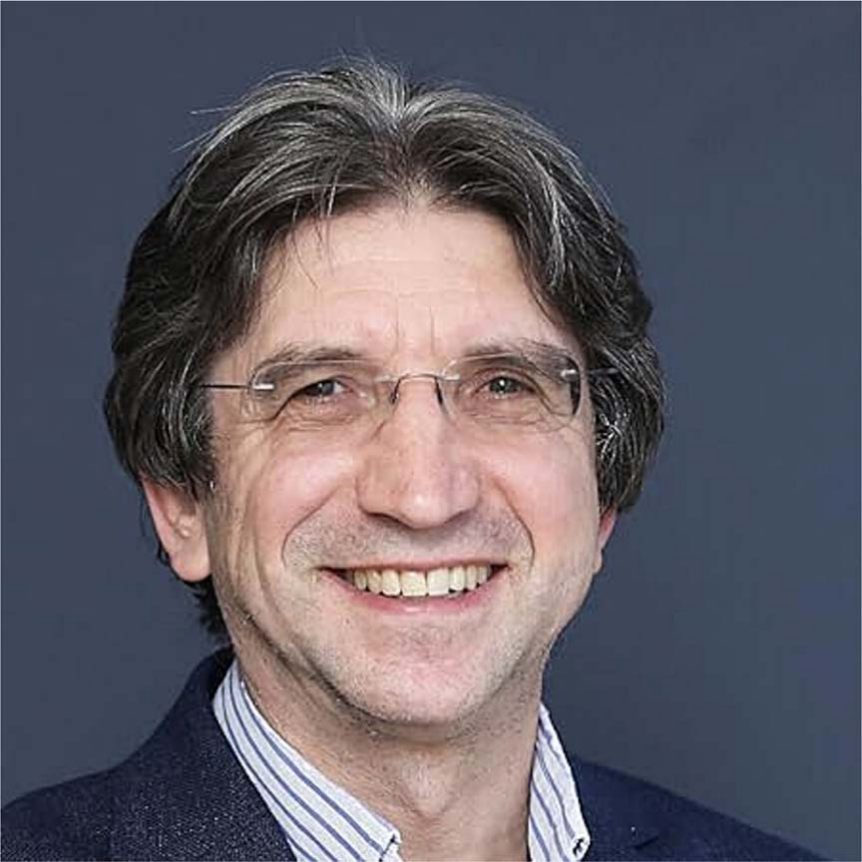


**17 SEPTEMBER 1962 TO 6 DECEMBER 2025**

With profound sadness we mark the passing of Professor Dan J. Stein—clinician, scientist, philosopher, and friend. A pioneer of International and African psychiatry, and a central figure in global neuropsychopharmacology, he devoted his life to understanding and relieving the burden of mental disorders in South Africa, across the African continent, and worldwide. In his death, we have lost a true giant.

## EARLY LIFE AND TRAINING

Dan Stein was born on September 17, 1962, in South Africa. He studied medicine at the University of Cape Town (UCT), completing an intercalated BSc degree with majors in biochemistry and psychology before graduating MB ChB with distinction.

Like many of his generation, he faced the moral crisis of apartheid. To avoid compulsory service in the all-White South African army, he left for the United States, where he completed residency training in psychiatry and a postdoctoral fellowship in psychopharmacology at Columbia University and the New York State Psychiatric Institute. He subsequently obtained two doctoral degrees, one in clinical neuroscience and another in philosophy from Stellenbosch University, reflecting a lifelong commitment both to rigorous science and to critical, reflective thinking about psychiatry.

When apartheid ended and Nelson Mandela was democratically elected President, Dan returned home. As he later reflected, this return was not only personal but also ethical: a commitment to help build a more just South Africa through science, clinical service, and public mental health. He remained based in South Africa for the rest of his life.

## BUILDING POSTAPARTHEID PSYCHIATRY IN SOUTH AFRICA

Soon after his return, Dan established what became the South African Medical Research Council (SAMRC) Unit on Anxiety & Stress Disorders, based at Stellenbosch University. Between 1994 and 2005, Dan directed the Unit at Stellenbosch University. The Unit, the first mental health unit of its kind in South Africa, undertook foundational work in anxiety neuroscience, initiated some of the country’s first brain MRI and neurogenetics studies in psychiatry, and led the South African Stress and Health (SASH) study, the first nationally representative survey of mental disorders on the African continent.

In 2017 this work evolved into the SAMRC Unit on Risk & Resilience in Mental Disorders, with Dan as Director and key partners at UCT, Stellenbosch University, and North-West University.

The Unit’s focus, spanning anxiety and stress, addictions, and other major mental disorders, epitomized his “bench, bed, bundu” philosophy: integrating laboratory neuroscience (“bench”), clinical research (“bed”), and public mental health and implementation science (“bundu,” the community).

From 2005, Dan served as Professor and Chair of the Department of Psychiatry and Mental Health at UCT. Under his leadership, psychiatric services, training, and research were strengthened across the Western Cape and beyond. He founded UCT’s Brain and Behaviour Initiative, which laid the foundation for the UCT Neuroscience Institute, where he became the inaugural Scientific Director of the first multidisciplinary neuroscience institute on the African continent.

His work on stigma, access to care, and the economic case for mental health helped reposition mental health in South Africa as both a human rights priority and a development imperative, contributing to incremental growth in provincial mental-health investments.

## LEADERSHIP IN AFRICAN PSYCHIATRY AND GLOBAL MENTAL HEALTH

Dan’s vision was never confined to one department or one country. He mentored generations of African clinicians and scientists whose work now spans addiction, child and adolescent psychiatry, liaison psychiatry, neurogenetics, neuroimaging, public mental health, psychopharmacology, and psychotherapy—many of them now leaders in their own fields.

He was a key architect of large collaborative projects that anchored African mental health in the global scientific conversation. The South African Stress and Health (SASH) study provided the first national prevalence data on common mental disorders in South Africa. The NeuroGAP-Psychosis collaboration brought together investigators in Ethiopia, Kenya, South Africa, and Uganda to address the profound underrepresentation of African genomes in psychiatric genetics, focusing on schizophrenia and bipolar disorder. He was also deeply involved in global collaborations such as the World Mental Health (WMH) surveys and the ENIGMA neuroimaging consortium.

Dan was deeply involved in the scientific community, such as the International College of Neuropsychopharmacology (CINP), European College of Neuropsychopharmacology (ECNP), and Scandinavian College of Neuropsychopharmacology (SCNP). Inspired by the CINP, he was the founding president of the African College of Neuropsychopharmacology (AfCNP), creating a home for African neuropsychopharmacology and helping to ensure that African perspectives and data shaped the global field. AfCNP congresses became a focal point for emerging African scholarship and collaboration.

## HONORARY PROFESSOR AND INTERNATIONAL COLLABORATOR

Dan’s impact extended well beyond Africa. He was a visiting professor at Mount Sinai School of Medicine in New York and at Aarhus University in Denmark. In Aarhus, he was appointed honorary professor in the Department of Clinical Medicine, Faculty of Health, where he worked to “build bridges between Aarhus and South Africa” and to advance translational psychiatry. He contributed to both research and teaching. He was celebrated in a large symposium that showcased his integrative approach to neuroscience and psychiatry.

## A SCHOLAR OF EXTRAORDINARY INFLUENCE

Dan’s scholarly output was astonishing in its breadth and depth. He authored or edited more than 50 books and more than 2000 journal articles and chapters, ranging from basic neuroscience to clinical trials, epidemiology, public mental health, and philosophy of psychiatry. Bibliometric analyses consistently place him among the most influential psychiatrists worldwide.

He was a Fellow of the African Academy of Sciences, Fellow of the Royal Society of South Africa, and Fellow of The World Academy of Sciences. He received several major international and national honors, including a Lifetime Achievement Award (WFSBP), Max Hamilton Memorial Award (CINP), John F. Herschel Medal (Royal Society of South Africa), and SAMRC Platinum Award for research excellence.

In *IJNP*, he played a key role in promoting evidence-based psychopharmacology, including leading an influential series in this journal, the *International Journal of Neuropsychopharmacology* (*IJNP*) and co-editing the book *Evidence-Based Psychopharmacology*, with Stephen Stahl and Bernard Lerer. He was a longstanding member of the editorial board of *IJNP*, where his perspective from low- and middle-income settings and his deep methodological rigor were of particular value.

Dan also played a critical role in psychiatric classification: he chaired the DSM-5 and ICD-11 workgroups on obsessive-compulsive and related disorders, helping to shape contemporary diagnostic frameworks that are now used worldwide.

Importantly, Dan was not only a prolific author and thoughtful editor but also an interlocutor: gently insisting that global psychiatry attend to African voices, African data, and African priorities. His latest book *Problems of Living: Perspectives from Philosophy, Psychiatry, and Cognitive-Affective Science* illustrates his integrative and thoughtful approach to mental and brain health.

His work on anxiety, obsessive-compulsive and related disorders, and trauma- and stressor-related disorders was at once biologically sophisticated and acutely attuned to social determinants and human rights. He showed that high-quality neuroscience and high-impact public mental health research are not luxuries reserved for wealthy countries, but necessities for societies emerging from colonialism, apartheid, violence, and inequality.

Dan’s research on the Truth and Reconciliation Commission focused on understanding the psychological makeup of perpetrators who committed human rights violations during apartheid. He examined how ordinary psychological processes, rather than clinical pathology, can contribute to participation in violence, emphasizing the roles of ideology, obedience, and group dynamics. His work highlighted the complex psychological mechanisms that allow individuals to commit atrocities while seeing themselves as morally justified. Overall, his analyses contributed to a deeper understanding of perpetrator psychology and informed broader conversations about reconciliation and accountability in postapartheid South Africa.

## COLLEAGUE, MENTOR, AND FRIEND

For all his extraordinary achievements, we who worked with Dan will remember above all his quiet generosity, intellectual humility, and unwavering collegiality. He answered emails from junior colleagues with the same care he gave to international committees; he read manuscripts with forensic attention but responded with warmth and encouragement.

As co-workers in the editorial world, we were privileged to experience his combination of absolute rigor and unfailing kindness. As an editor, reviewer, and author, he consistently strengthened our work and our field. As a friend, he brought wisdom, humor, and a steady ethical compass to every conversation.

## A LOSS BEYOND MEASURE

Professor Dan J. Stein’s far too early death in December 2025, at the age of only 63, leaves an enormous void in African psychiatry, in global neuropsychopharmacology, and in the lives of the many colleagues, mentees, and friends who loved and admired him.

Dan returned to South Africa when apartheid fell, and he spent the rest of his life building. Building services, building institutions, building science, building the careers of others, and building bridges between Africa and the world. His work will continue in the departments he led, the units he founded, the colleges and journals he served, the students he taught, and the lives of the patients whose suffering he helped to relieve.

In mourning his passing, we also honor his example: integrative in thought, principled in action, and generous in spirit.

Our sincere thoughts are with his wife, Professor Heather Zar, children Gabriella, Joshua, and Sarah, and grandson, Rafa, to whom we extend our deepest condolences and enduring respect.

Gregers Wegener: Editor-in-Chief, IJNP; Past President, SCNP

Bernard Lerer: Founding Editor-in-Chief, IJNP

Alan Frazer: Past Editor-in-Chief, IJNP; Past President, ACNP

Anthony Grace: Past Editor-in-Chief

Kazutaka Ikeda: President, CINP; Vice President AsCNP

Gabriella Gobbi: President Elect, CINP

Allan Young: Vice President, CINP; Past President, BAP

Joseph Zohar: Past President, CINP; Past President, ECNP

Pierre Blier: Past President, CINP

Siegfried Kasper: Past President, CINP; Past President, WFSBP

John Krystal: Past President, ACNP; Past President, CINP

Shigeto Yamawaki: Past President, CINP

Anthony Philips: Past President, CINP

Hans-Jürgen Möller: Past President, CINP; Past President, WFSBP; Past President, EPA

Robert H. Belmaker: Past President, CINP

Lukoye Atwoli: Past President, AfCNP

David Nutt: Past President, BAP; Past President, ECNP

Brian H. Harvey

Soraya Seedat: President Elect, WFSBP

Michael Berk: Vice President, WFSBP

Robin Emsley

## ACRONYMS

- ACNP: American College of Neuropsychopharmacology
- AfCNP: African College of Neuropsychopharmacology
- AsCNP: Asian College of Neuropsychopharmacology
- BAP: British Association for Psychopharmacology
- CINP: International College of Neuropsychopharmacology
- ECNP: European College of Neuropsychopharmacology
- EPA: European Psychiatric Association
- IJNP: International Journal of Neuropsychopharmacology
- SCNP: Scandinavian College of Neuropsychopharmacology
- WFSBP: World Federation of Societies of Biological Psychiatry

## Data Availability

Data availability is not applicable as no new data were created or analysed.

